# Improving and evaluating deep learning models of cellular organization

**DOI:** 10.1093/bioinformatics/btac688

**Published:** 2022-10-20

**Authors:** Huangqingbo Sun, Xuecong Fu, Serena Abraham, Shen Jin, Robert F Murphy

**Affiliations:** Computational Biology Department, Carnegie Mellon University, Pittsburgh, PA 15213, USA; Department of Biological Sciences, Carnegie Mellon University, Pittsburgh, PA 15213, USA; Computational Biology Department, Carnegie Mellon University, Pittsburgh, PA 15213, USA; Computational Biology Department, Carnegie Mellon University, Pittsburgh, PA 15213, USA; Computational Biology Department, Carnegie Mellon University, Pittsburgh, PA 15213, USA

## Abstract

**Motivation:**

Cells contain dozens of major organelles and thousands of other structures, many of which vary extensively in their number, size, shape and spatial distribution. This complexity and variation dramatically complicates the use of both traditional and deep learning methods to build accurate models of cell organization. Most cellular organelles are distinct objects with defined boundaries that do not overlap, while the pixel resolution of most imaging methods is n sufficient to resolve these boundaries. Thus while cell organization is conceptually object-based, most current methods are pixel-based. Using extensive image collections in which particular organelles were fluorescently labeled, deep learning methods can be used to build conditional autoencoder models for particular organelles. A major advance occurred with the use of a U-net approach to make multiple models all conditional upon a common reference, unlabeled image, allowing the relationships between different organelles to be at least partially inferred.

**Results:**

We have developed improved Generative Adversarial Networks-based approaches for learning these models and have also developed novel criteria for evaluating how well synthetic cell images reflect the properties of real images. The first set of criteria measure how well models preserve the expected property that organelles do not overlap. We also developed a modified loss function that allows retraining of the models to minimize that overlap. The second set of criteria uses object-based modeling to compare object shape and spatial distribution between synthetic and real images. Our work provides the first demonstration that, at least for some organelles, deep learning models can capture object-level properties of cell images.

**Availability and implementation:**

http://murphylab.cbd.cmu.edu/Software/2022_insilico.

**Supplementary information:**

Supplementary data are available at *Bioinformatics* online.

## 1 Introduction

Deep learning has been very successfully used in recent years for biomedical image analysis applications, including for analysis and modeling of fluorescence microscope images. Deep learning applications to fluorescence microscope images have included reconstruction of super-resolution images (Ouyang *et al.*, 2018), cell segmentation (Greenwald *et al.*, 2022; Stringer *et al.*, 2021), integrative tissue analysis (Bao *et al.*, 2022; Maric *et al.*, 2021) and augmented microscopy (Wang *et al.*, 2021).

Despite its power, there are significant limitations of fluorescence microscopy for observing and modeling the complex variation in number, size, shape and spatial distribution of subcellular structures. One is the limited spatial resolution that makes it difficult to resolve individual organelles. Another is the limited number of fluorescent tags that can be observed simultaneously. While this can be partially overcome by multiplexing in fixed cells (Goltsev *et al.*, 2018; Lin *et al.*, 2015; Schubert *et al.*, 2006), it limits learning of complex relationships between multiple structures in live cells.

Nonetheless, learning and modeling these relationships is an important challenge in cell biology. Initial work using traditional computer vision methods introduced the idea of building generative models of cells in which organelle positions within cells were conditional upon other parts, such as the cell membrane, nuclear membrane and microtubules (Johnson *et al.*, 2015; Majarian *et al.*, 2019; Zhao and Murphy, 2007). With the advent of deep learning and the creation of large collections of images labeled for specific organelles, a significant advance occurred through the creation of autoencoder models for organelles that were also conditional on cell and nuclear membranes (Donovan-Maiye *et al.*, 2022).

This conditional approach has been taken even further by constructing deep learning models that are conditional upon an easily acquired common reference image, such as a transmitted light image. In this ‘*in silico* labeling’ or ‘label-free microscopy’ approach, separate deep neural networks are trained to predict the likely distribution of specific organelle markers from transmitted light or other label-free images. Christiansen *et al.* (2018) first proposed this method to label the nucleus and neurons; Ounkomol *et al.* (2018) dramatically extended this approach by *in silico* labeling of multiple subcellular structures; further enhancements have been described (Cooke *et al.*, 2021; Waibel *et al.*, 2019). These approaches used variations on the powerful U-Net model (Falk *et al.*, 2019; Ronneberger *et al.*, 2015). Wang *et al.* (2021) described an improved U-Net architecture which inserted a self-attention module into the U-Net down-sample module to help enlarge the receptive field of the model. Nonetheless, as with other autoencoder-based models, these implementations are trained with only a pixel-wise loss function (like mean squared error, MSE) which often results in their producing relatively blurred images (Huang *et al.*, 2018). These may not accurately reflect the edge morphology of subcellular structures, especially for smaller objects. One alternative is to modify the convolutional neural network (CNN) architectures. Deep Recurrent Attentive Writer (DRAW) (Gregor *et al.*, 2015) was proposed to generate realistic images using a variational autoencoder (VAE) with recurrent blocks. The subsequent convolutional DRAW (Gregor *et al.*, 2016) further combined the recurrent blocks with convolution components to improve the model. Another alternative is to modify the loss function used in training. PixelRNN and PixelCNN are well-known generative models, which employ an auto-regressive method to learn the explicit distribution (Van den Oord *et al.*, 2016a; Van den Oord *et al.*, 2016b). Lastly, Generative Adversarial Networks (GAN) have been shown to generate sharper, more realistic instances (Goodfellow *et al.*, 2014).

‘Pix2Pix’ network is a GAN variant that combines a U-Net-based generator and a PatchGAN discriminator (Isola *et al.*, 2017). Experiments showed that the Pix2Pix network performs well on image-to-image translation. Predicting fluorescence or stain distributions is also an image-to-image translation problem, and prior work such as Rivenson *et al.* (2019) has used a variant of the pix2pix network for virtual tissue staining.

While previous work has shown promising results, there is significant room for improvement, especially for *in silico* labeling of multiple cellular structures. One potential direction is to design more efficient model architectures, and another is to set up novel model-training approaches and objectives specialized for this task. The current state-of-the-art approaches treat each protein channel (subcellular structure) independently by building separate models from the reference input. These essentially give the probability of each pixel being in a given structure (or equivalently the amount of each structure expected to be present in each pixel), but do not address any limitations on whether more than one structure can be in the same pixel. In other words, they do not address the concept of exclusivity of structures, which, while complicated by the pixel resolution, is expected for most membrane-bound organelles. Depending on pixel resolutions, a single pixel may cover boundaries of two organelles. Nonetheless, a low fraction of pixels with overlapped organelles is still expected. Further, most current supervised learning-based computer vision approaches applied in biological studies only rely on pixel-wise evaluations, which do not consider the number and shape of individual biological components nor their spatial distributions. Lastly, while there is a large body of work on designing generative models, there is only limited work focused on criteria for evaluating generative models (Barratt and Sharma, 2018; Szegedy *et al.*, 2016). Therefore, higher-order metrics can be useful to better understand the quality of generative models.

**Fig. 1.**
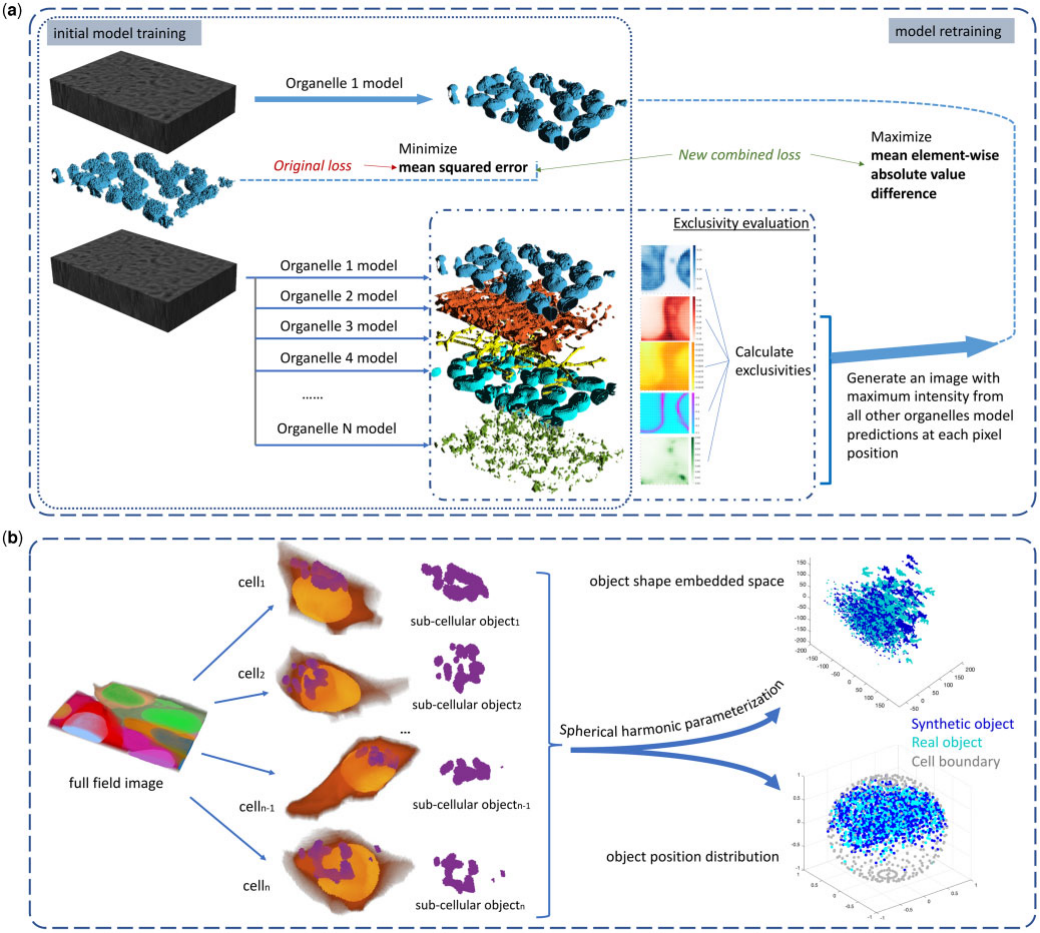
Approaches to evaluating and improving organelle generative models. (**a**) The model training process described by Ounkomol *et al.* (2018) is shown on the left and the process of evaluating overlap between predicted organelles and retraining models to reduce it is shown on the right. After training individual organelles models, each model was retrained in sequence using the additional loss term that seeks to minimize overlap (maximize exclusivity). (**b**) The process for object-based evaluation of synthetic images using CellOrganizer is illustrated. For each organelle model, real and synthetic images are segmented into individual objects. The real and synthetic object shapes are described using spherical harmonic parameterization and displayed in a 3D embedding, and their positions normalized to a spherical cell are also shown. The distributions of real and synthetic objects in both shape and position are compared statistically as described in the Section 2

In this work, we describe a 3D Pix2Pix network (Isola *et al.*, 2017) containing recurrent residual convolutional units described in Alom *et al.* (2019). Without increasing the size of the network, recurrent units allow a deeper network. The residual-like block also gains the advantage of ResNet to maintain the deep network (He *et al.*, 2016). Then, we developed novel evaluation metrics for multi-channel reconstructed fluorescence images. The first type was based on ‘exclusivity’, which describes the self-consistency of a predicted image containing multi-subcellular organelles under the assumption that overlap between those organelles should be minimal. We showed efficient ways to improve the exclusivity of *in silico* labeled images by using both modified loss functions with an additional ‘exclusivity’ term and novel neural network architectures compared to the previous work by Ounkomol *et al.* (2018). The second metric type was derived from object-wise evaluations of subcellular structures. We compare object shapes between real and synthetic images by using spherical harmonic parameterization, which has been shown to provide better performance at representing the shape of objects like cells and nuclei (Ruan and Murphy, 2019; Styner *et al.*, 2006). An overview of the approaches used for evaluating and improving generative models of organelles is provided in Figure 1.

## 2 Materials and methods

### 2.1 Image dataset

We performed our evaluations using the Allen Institute for Cell Science image dataset that was used by Ounkomol *et al.* (2018). It contains fluorescence microscope images for 12 different subcellular components (actin filaments, microtubules, endoplasmic reticulum, desmosomes, cell membrane, actomyosin bundles, Golgi apparatus, DNA, nuclear envelope, mitochondria, nucleoli and tight junctions) and their corresponding transmitted light images. For each subcellular component, we divided the dataset into 60 images for training and 20 images for testing.

### 2.2 Deep neural network architecture and training and validation

We implemented three deep neural network architectures for training organelle models. The first is the same as the one proposed in Ounkomol *et al.* (2018), which we will refer to as the U-Net model for simplicity. For each organelle, the U-Net model was trained for 100 000 iterations.

The second is a model adapted from the original Pix2pix network model, which we refer to as a Vox2Vox network with recurrent units (Vox2Vox-RU). This model contains two parts: a discriminator with a stack of 3D convolutional layers, and a generator modified from the 3D version of recurrent residual convolutional neural network based on U-Net (R2U-Net) (Alom *et al.*, 2019). The essential idea in this model is to replace the convolutional layers by recurrent residual convolutional units (RRCU) in the U-Net. RRCU consists of a 1 × 1 convolution layer and 2 subsequent recurrent convolutional layers (see Figs 3 and 4 in Ounkomol *et al.*, 2018), which increases the model depth without adding new parameters but also adopts the idea from ResNet to mitigate limitations of deep net training. The detailed architecture of this model can be found in Supplementary Figure S1.

The third is a 3D Pix2Pix model whose generator is the same as the U-Net and whose discriminator is the same as shown in Supplementary Figure S1.

Both 3D Pix2Pix and Vox2Vox-RU models were trained for a total of 20 000 iterations with an Adam optimizer with a learning rate of $2\times 10^{-4}$ and a batch size of 4. To provide a ‘warm start’ in order to maintain stable training, for the first 2500 iterations the value of real and fake labels provided for the discriminators were set to

$$\{\begin{array}{cc} 1-\left(0.02\times (5-\left\lfloor i/500\right\rfloor )\right)\times N(0,1) & \text{real label}\\ 0+\left(0.02\times (5-\left\lfloor i/500\right\rfloor )\right)\times N(0,1) & \text{fake label}, \end{array}$$

where *i* refers to the iteration number. The soft labels prevent the discriminator from learning much faster than the generator which can lead the network to fail to converge.

After initial training of all organelle models by both methods, we predicted all organelle signals with the trained 12 models for all transmitted light images for both training approaches.

### 2.3 Evaluation of organelle exclusivity

We defined three measures of organelle ‘exclusivity’. All used the average over some set of pixels between the highest predicted value and the second highest predicted value. Overall exclusivity was defined as this value for all pixels. Organelle exclusivity was defined for each organelle individually as the average only for pixels for which that organelle has the highest predicted value. Pairwise exclusivity was defined for each directed pair of organelles as the average only for pixels for which the first organelle has the highest predicted value of the difference between that predicted value and the predicted value for the other organelle (thus it is not symmetric).

### 2.4 Retraining

After training the original models, we implemented a new loss function to enlarge the exclusivity. We retrained all the models in the reverse order of the initial organelle exclusivity. The new loss function adds a term which maximizes the mean element-wise absolute value difference between the prediction of the current model and the maximum intensity at each pixel position of all other subcellular component predictions. The principle is that if the organelle model we are training has the highest intensity at a pixel position, then we want to enlarge the differences between the pixel intensity of that organelle and the second highest pixel intensity among the remaining organelles; if the trained organelle is not the highest intensity at a pixel position, then we want it to be as small as possible compared with the organelle with the largest intensity.

The U-Net model was fine-tuned with 12 500 additional iterations for retraining. We used a parameter, *p*, to control the extent to which the exclusivity is weighted in the loss function. We scanned through a potential list of candidate values of *p* and selected the value that gave the highest ratio of overall exclusivity to overall MSE for each model. We updated the predictions after each subcellular component model was retrained, and moved on to retrain the next subcellular component. For retraining the Vox2Vox-RU model, to better balance the three loss terms (GAN loss, MSE/L1 loss, exclusivity loss) and reduce computation time, the weight of exclusivity loss in Vox2Vox-RU was set manually.

(1) $$\text{Retrain Loss}=\{\begin{array}{cc} \text{MSE}+p\cdot \text{Exc}.\text{Loss} & U-\text{Net}\\ \text{GAN Loss}+\lambda \cdot \text{MSE}+p\cdot \text{Exc}.\text{Loss} & \text{Vox}2\text{Vox-RU} \end{array}$$

The loss function for training Vox2Vox-RU has an additional L2 or L1 loss term (which differs for different organelles) between the prediction and real fluorescence tag which helps to further stabilize the training process; the detailed loss functions used are reported in Supplementary Table S1. The different loss function terms were manually set mainly based on the texture of subcellular patterns. For example, for tubule-like structures, since the training image resolution is limited (which makes tubule-like structure patterns blurry), we used L2-loss; for membrane-like structures, to maintain a thin reconstructed structure, we used L1-loss.

Unlike U-Net retraining, Vox2Vox-RU was retrained from scratch with the same optimizer for 25 000 iterations, still with a warm start.

### 2.5 Subcellular object shape and spatial distribution analysis

Shape representation with spherical harmonic transforms uses a series of orthogonal functionals to fit the surface of a genus-0 topology object, providing an efficient way to parameterize objects (Nain *et al.*, 2007; Ruan and Murphy, 2019). The quality of spherical harmonic parameterization can be assessed by calculating the Hausdorff distance between the original shape’s surface and the reconstructed shape’s surface. The quality of spherical harmonic parameterization heavily depends on the order of spherical harmonics; we set it to 31 in this work.

To compare the synthetic images from DL models and real images, we segmented individual cells and the organelles within them. Nuclear and cell boundaries were found using the Segmenter Model Zoo (https://github.com/AllenCell/segmenter_model_zoo). Individual organelles were segmented using the Allen Cell & Structure Segmenter (https://www.allencell.org/segmenter.html) with default settings. These tools were specifically developed using the image sets used in our study. Partial cells at the boundary of the images as well as the objects in those cells were discarded, since organelle shapes and positions could not be accurately determined for those cells. The organelle objects were parameterized via spherical harmonic transform. To determine whether the objects from the synthetic images were comparable in spherical harmonic descriptor space, principle component analysis was performed to reduce the dimensionality of the spherical harmonic descriptors (we used the first three components). Given two populations of low-dimensional shape descriptors $x_{1},\ldots ,x_{m},x_{m+1},\ldots ,x_{m+n}$, where *m* denotes the number of objects in synthetic images and *n* denotes the number of objects in real images, we give them labels to indicate the population they come from as $y_{1}\ldots ,y_{m}=1$ and $y_{m+1},\ldots ,y_{n}=2$. Inspired by the k-clique percolation approach (Palla *et al.*, 2005), for each object, we find the purity of its *k* nearest neighbors objects. Ideally, if two populations merge well, the label fraction will be close to $\frac{m}{m+n}$, and if two population lay separately in the spherical harmonic descriptor space, the purity of neighbor objects will be higher. Formally, we calculate

(2) $$\text{score}=\frac{1}{m+n}\left(\sum_{i=1}^{m+n}p_{i}\;\text{log}\;\frac{p_{i}}{q}+(1-p_{i})\;\text{log}\;\frac{1-p_{i}}{1-q}\right),$$

which is the average KL-divergence between the neighbor objects purity $p_{i}=\frac{\sum_{j|x_{j}\in N(x_{i})}I(y_{j}=1)}{k}$ and reference purity $q=\frac{m}{m+n}$, where N(·) returns the *k*-nearest neighbor object set and I(·) refers to the indicate function. For this analysis, very small values of *k* will in general result in larger divergences even for similar populations as they are more sensitive to local variations. In contrast, very large values will decrease the divergence (and the sensitivity of the test) even for different populations since they will encompass large fractions of both populations. We calculated divergences for different *k*.

To compare the spatial distribution of subcellular components inside the cell, we first align all valid cells by their major axis, then map the cell boundary to a unit sphere and the nucleus to the center of the sphere. All the centers of objects in the cell are mapped onto these polar coordinates, and a Gaussian kernel is used to generate a continuous object distribution in the unit sphere domain. The KL-divergence was then calculated between this fit for synthetic images and real images.

### 2.6 Availability and implementation

The images used are available from https://downloads.allencell.org/publication-data/label-free-prediction/. The analysis of organelle shape and spatial distribution was performed using version 2.9.3 of the open source CellOrganizer system (http://CellOrganizer.org, https://github.com/murphygroup/cellorganizer). A Reproducible Research Archive containing all source code, generated images and analysis results is available at http://murphylab.cbd.cmu.edu/Software/2022_insilico.

## 3 Results

### 3.1 Comparing organelle model learning approaches

**Fig. 2.**
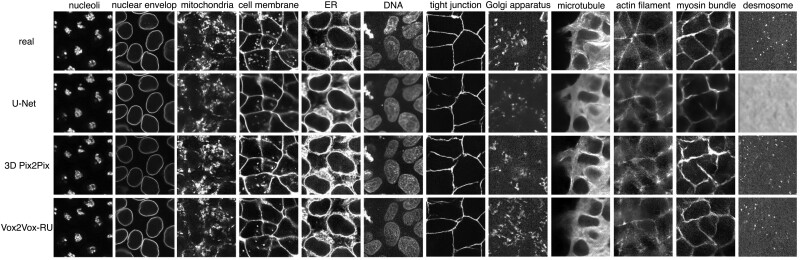
Example real and predicted images

We began by training individual organelle models with U-net, 3D Pix2Pix and our new approach. We used the same dataset described by Ounkomol *et al.* (2018), and randomly divided it into training and test sets. Figure 2 shows a comparison between predictions from the U-Net, 3D Pix2Pix and Vox2Vox-RU models for nine different organelles. When examined visually, all three of the models give reasonable predictions compared to the real images, although the 3D Pix2Pix and Vox2Vox-RU images are closer to the real images in terms of preservation of contrast level, detailed texture and sharpness of organelle boundaries. This difference is most pronounced for the desmosome model. When evaluated by the overall mean-square error (MSE) between the original and predicted images, most U-Net models perform better than 3D Pix2Pix and Vox2Vox-RU models (Supplementary Table S2). However, the U-Net desmosome model has a very high MSE, as may be expected given the visual difference. The 3D Pix2Pix and Vox2Vox-RU models perform reasonably for all organelles such that their overall MSE is lower, but this is reversed when the desmosome model is removed from the averages. These results are consistent with the difference in goals of the two modeling approaches: the GAN-based models will produce models with better ‘high level’ performance than U-Net models at the sacrifice of a small increase in MSE.

**Fig. 3.**
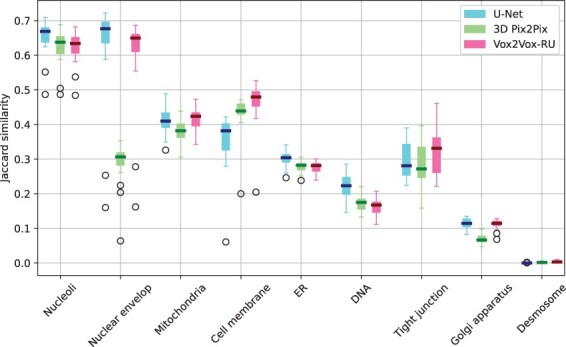
Image-wise Jaccard similarities for segmented images produced by different models compared to the segmented real images. The short line in the center of each box refers to the median value

We therefore sought to quantitatively evaluate the performance of the three modeling approaches from the ‘high level’ viewpoint of subcellular organelle morphology. To do this, we performed segmentations of original and synthetic images using the Allen Cell & Structure Segmenter (see Materials and methods). We then calculated the Jaccard similarity between the segmentations for all three approaches (Fig. 3). (Since we observed that the segmentation results for the tubular organelles microtubule, actin-filaments and actomyosin were poor, we did not include these organelles in the comparison.)

All three modeling approaches performed similarly for most of the organelles. Both U-Net and Vox2Vox-RU performed best for the relatively easy task of generating nuclear components (nucleoli and nuclear envelope), whereas 3D Pix2Pix had comparable results for nucleoli but poor results for nuclear envelope. It is worth noting that Vox2Vox-RU significantly outperforms U-Net in cell membrane prediction, with a roughly 10% higher median value and 3D-Pix2Pix falls in between; this is consistent with the observation in Figure 2 that the U-Net model produces thicker cell membranes than those in the original image. Also for nuclear envelope, mitochondria and Golgi apparatus, 3D Pix2Pix has inferior performance compared with other two models, and did not achieve top performance for any organelle. Note that these results evaluate *semantic* segmentation which simply seeks to distinguish organelle foreground from cell background and does not consider *instance* segmentation which evaluates individual organelle morphology. We will consider this later. The Vox2Vox-RU outperforms 3D Pix2Pix in Jaccard similarity on 6 out of 9 organelles and almost ties on the rest. This suggests that the generator in Vox2Vox-RU is better for GAN-based training.

### 3.2 Exclusivity analysis and retraining

**Fig. 4.**
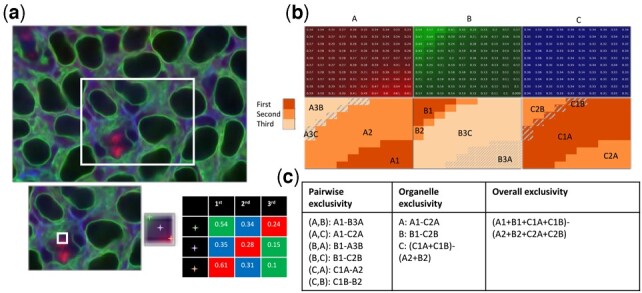
An illustration of different metrics for exclusivity. (**a**) An example of a combined image showing the values of three organelle predictions using red, green and blue. The insets show magnification of smaller and smaller regions. (**b**) Predictions for the smallest region in (a). The first row shows the intensity of each organelle channel (A, B, C). The second row shows the pixel-wise intensity relationships. The darkest region indicates that the channel in that column has the highest intensity among all three at that position, the lighter region indicates that the channel has the second highest intensity, and the lightest region indicates that the channel has the lowest intensity. The code in each subregion summarizes its properties: the first letter and number indicate the ranking of that channel in that subregion, and the second letter (if present) indicates which channel is highest in that subregion. (**c**) A detailed example of the definitions for the different exclusivity measure. The use of a code in the definitions represents the pixel-wise operations corresponding to that code (‘–’ is pairwise subtraction and ‘+’ is pairwise addition)

Both the training and retraining processes of U-Net and Vox2Vox-RU models are carried out separately for each organelle, and therefore, when the resulting models are used to make predictions for a given transmitted light image, their predictions are independent. In other words, an intensity level is predicted for each pixel for each organelle, and it is entirely possible that high intensity values will be predicted for the same pixel for more than one organelle. Such ‘overlap’ between organelles in the same pixel can result from the two organelles having similar characteristics in transmitted light images, or from the learned models having significant blur or uncertainty around the edges of predicted organelles. The first may be unavoidable, but the second can be a reflection of the quality of the training. We therefore propose new criteria for evaluation of label-free microscopy—organelle ‘exclusivity’. The premise is that most pixels in an image should have only one organelle with high predicted content. This concept can be quantified for all organelles, each organelle individually relative to all others, and each organelle relative to each other organelle (Fig. 4).

The organelle-specific exclusivities for all three models, as well as the overall exclusivities, are reported in Table 1. Our proposed Vox2Vox-RU model reaches a higher overall exclusivity compared with U-Net, and 3D Pix2Pix falls in between. Some organelles such as nucleoli and nuclear envelopes have high exclusivity and low MSE for both U-Net and Vox2Vox-RU (Supplementary Table S2). On the other hand, microtubules, actin filaments and endoplasmic reticulum have both low MSE and low organelle exclusivity, suggesting that despite the accuracy of the prediction compared with the true protein signals, the models are limited in their ability to distinguish between them. As noted above, the desmosomes U-Net model performs poorly in MSE due presumably to their small sizes, yet the Vox2Vox-RU and 3D Pix2Pix models are still able to produce predictions with relatively reasonable MSE. All desmosome models have higher exclusivity than microtubules, actin filaments and ER, indicating that they are better distinguished than those organelles.

**Table 1.** The organelle exclusivity (see Fig. 4 for the definition) on the test set

	U-Net	3D Pix2Pix	Vov2Vox-RU
Microtubule	0.409	0.560	0.57
Actin filament	0.401	0.527	0.577
Desmosome	0.526	0.61	0.659
DNA	1.05	0.892	0.86
Nucleoli	1.50	1.931	1.69
Nuclear envelope	1.11	0.966	1.11
Cell membrane	0.756	1.009	1.05
Actomyosin bundle	0.738	1.003	0.807
Endoplasmic reticulum	0.433	0.565	0.58
Golgi apparatus	0.774	0.698	0.767
Mitochondria	1.41	1.180	1.2
Tight junction	1.50	1.007	1.04
Overall	0.7930	0.847	0.8635

*Note*. Note that exclusivity values are in units of pixel intensities.

The overall exclusivity of trained 3D Pix2Pix models is intermediate (0.8470), which further confirms that our improved results Vox2Vox-RU are due both to the use of GAN training and the architecture change in the generator. Comparing the organelle exclusivity values with the mean square errors, we see little correlation for the U-net models (R2=–0.03) and mild negative correlation for the Vox2Vox-RU models (R2=–0.38). This reinforces the conclusion from visual analysis that the GAN training can capture both aspects, and strongly indicates the value of not evaluating organelle model methods using MSE alone.

**Fig. 5.**
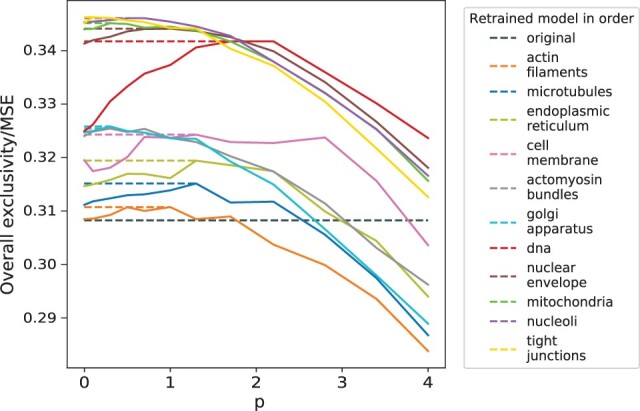
The improvement trajectories of the ratio of overall exclusivity and MSE through retraining of different protein models using U-Net. The weight *p* was scanned from 0 to 4 and the *p* resulting in the maximum ratio of overall exclusivity and MSE was chosen. The results for that organelle were replaced with the optimal model and used in retraining the next organelle model

In view of the large overlap for some organelle models, we next explored whether we could retrain the U-net and Vox2Vox-RU models to increase the exclusivity. To do this, we added an additional term to the loss function so that learning would balance the original goal with increasing exclusivity. We used a greedy approach to improve overall exclusivity, retraining each organelle model in the order of decreasing exclusivity (see Eq. 1). However, a critical issue was how to weight the contribution of exclusivity to the overall loss. When the parameter *p* is 0, the loss function degenerates to MSE, and exclusivity is not considered. When *p* is larger, the predicted organelles tend to overlap less (larger exclusivity) but deviate more from their original intensities (larger MSE). The effect can be different for different organelles. Therefore, for the U-net model (as described in Section 2), for a given organelle, we varied *p* and chose the value that would optimize the ratio of the resulting overall exclusivity and MSE (Fig. 5). This was chosen for each organelle in turn; the model for that organelle was then replaced with the model for the chosen *p*.

All models were retrained except desmosome (since the large MSE after the original training process compared to other organelles would inappropriately bias retraining toward extremely large exclusivity when using the ratio criterion). As can be seen, the overall exclusivity gradually rose as each model was retrained, and the optimal weights for the different organelles were roughly similar. When retraining the Vox2Vox-RU models, we did not do a grid search over weights but rather manually assigned a fixed weight for a given organelle; this was because of the high computational cost for grid search and potential difficulty maintaining a stable training process (see Supplementary Table S1). After the whole retraining process, the overall exclusivity increased about 24 and 13.17% for U-Net and Vox2Vox-RU models on the test set. The smaller improvement on Vox2Vox-RU models could be due to the optimization of the loss term for the U-Net but not the Vox2Vox-RU models. Example fluorescence tag predictions from the retrained models are shown in Supplementary Figure S2.

**Fig. 6.**
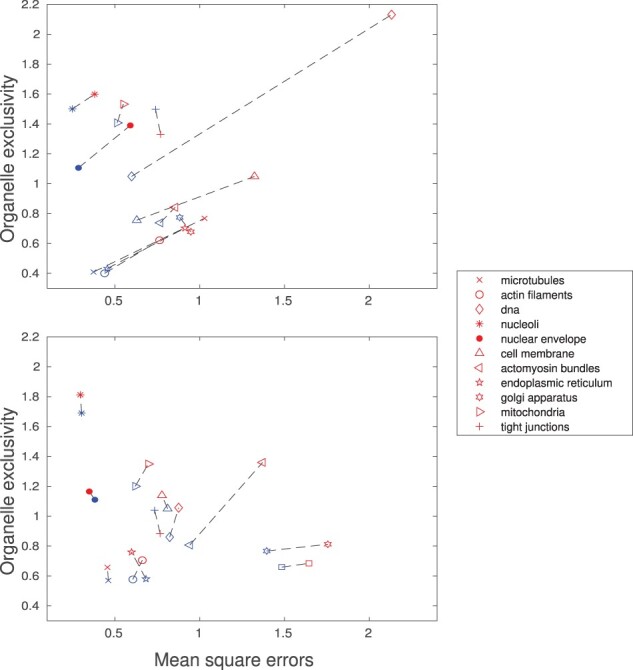
The MSE and organelle exclusivity before (blue) and after (red) the retraining for U-Net (upper) and Vox2vox (bottom), connected by dashed lines for specificity. (A color version of this figure appears in the online version of this article)

Comparisons of organelle exclusivity and MSE before and after the retraining for both models are shown in Figure 6. Overall, for both models, most organelles sacrifice a slight increase in MSE to get a fair increase in organelle exclusivity. The improvement in pairwise exclusivity upon retraining is generally consistent with the changes in organelle exclusivity, as shown in Supplementary Figure S3. We also record changes of each organelle’s exclusivity during retraining and typically see a slight decrease in exclusivity of other organelles after a specific organelle model is retrained (Supplementary Fig. S4).

### 3.3 Subcellular component shape and spatial distribution analysis

The criteria we have described so far measure reconstruction error both at the level of individual pixels and at the level of segmentation into regions above and below a threshold. The exclusivity metric we have introduced sought, in part, to evaluate a different aspect, the sharpness of the boundaries between predicted organelles. Since most of the models we have evaluated are for organelles that consist of distinct objects, we next sought to evaluate whether those models could properly capture the number, shape and cellular positions of those organelles. We segmented all real and synthetic images into individual cells (ignoring incomplete cells that extend beyond the boundary of the image) and then segmented individual organelles (see Section 2). This yields a set of objects for each complete cell for each image for each organelle. Each object was represented by a list of the pixels contained within it and by the coordinates within the cell of its centroid. To evaluate the consistency between the object shapes from real and synthetic images, we chose, based upon our prior work on evaluation of models of cell and nuclear shape, to use spherical harmonic (SPHARM) parameterization to construct object shape models. Our previous results showed that our modified implementation of SPHARM modeling performed better than other available methods (including deep learning methods) for modeling even eccentric 3D cell shapes. However, this parameterization approach only applies to genus-0 objects (those without holes), and we therefore only created object-based models for mitochondria, nucleoli, Golgi apparatus and desmosome.

Each object is described by a high-dimensional shape vector that can be converted (back-transformed) into a shape. We can then create a generative model of object shape by choosing a lower dimensional embedding of the vectors. To confirm that each object’s shape is being accurately represented by the SPHARM parameterization, we first calculate the Hausdorff’s distance between the original object and its reconstruction from the lower dimensional embedding (Hausdorff’s distance is the largest difference between the locations of equivalently spaced points on two shapes). As shown in Supplementary Table S3, most of the objects are well-fitted with average Hausdorff distance around 1 µm. We also observe that the number of objects produced by both models is comparable to the real images.

Given two sets of objects (e.g. from synthetic and real images), we can construct a lower-dimensional embedding of the object shapes using just the first three principal components of the SPHARM vectors of both sets. The two sets can be visually compared in plots in which downsampled versions of each shape are displayed at their position in the 3D embedding (Supplementary Figs S5–S8).

To make the comparison quantitative, we compared the distributions of the two sets in the reduced spherical harmonic descriptor space. As described in Section 2, the principle is that the similarity of the object distributions can be compared by measuring the fraction of the neighbors of each object that are real objects. We calculate this as the KL-divergence (Eq. 2) between the observed fraction and the expected fraction based upon the number of objects from each type of image. This was calculated for various numbers of neighbors (see Supplementary Table S4).

To compare the spatial distributions of objects within the cell, we converted each object’s Cartesian coordinates within the image into a common frame of reference. We chose a spherical cell as this reference and mapped each object’s centroid into polar coordinates in which the radius is the fractional distance of the object from the cell membrane. The distributions for real and synthetic objects can be visually compared in Supplementary Figures S9–S12. We also calculated the KL-divergence between the two probability densities (Supplementary Table S5).

**Fig. 7.**
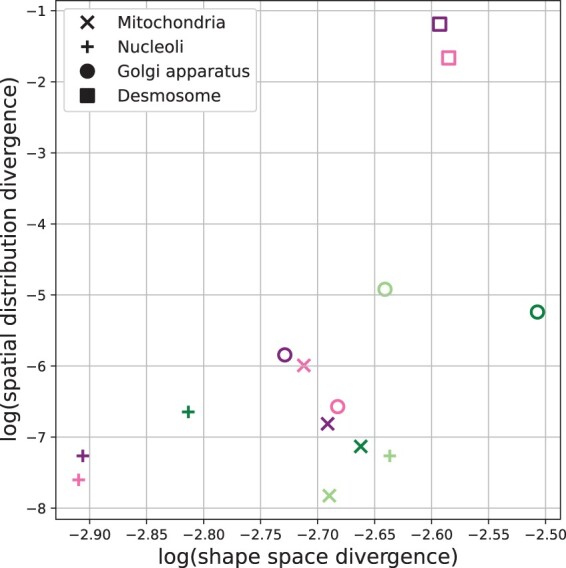
Models’ logarithmic divergence in spherical harmonic descriptor shape space (*x*-axis) and cellular spatial distribution (*y*-axis). The pink and purple markers refer to the original and retrained Vox2Vox-RU model, respectively. The light green and dark green markers refer to the original and retrained U-Net model, respectively. Shape divergence is for *k *=* *8. (A color version of this figure appears in the online version of this article)

Figure 7 shows a scatter plot of the two divergences versus each other, in which low values for both (the lower left corner) correspond to similar spatial and shape distributions. Both models perform well on nucleoli (especially after retraining), which can be expected since the shape of nucleoli is relatively regular and they are located in the relatively small nuclear which leads to a very small spatial distribution divergence for both models. The Vov2Vox-RU model outperforms the U-Net model on the Golgi apparatus and does slightly better on mitochondria. This is consistent with our observation in Figure 2 that Vov2Vox-RU model produces objects with sharper edges and appearances more similar to the objects in the real images. In contrast, modeling the desmosome is the hardest task for both models, since it is small in size and distributed throughout the entire cellular environment. The U-Net models fail to produce reasonable desmosome objects, and the Vox2Vox-RU model produces desmosome objects which are comparable to the real objects but fails to accurately recover its spatial distribution.

To estimate the statistical significance of the divergences, we performed a permutation test to establish the divergence expected between objects from two different sets of real images. The *P*-values are also shown in Supplementary Tables S4 and S5. Large *P*-values indicate that we cannot reject the null hypothesis that the synthetic and real images are drawn from the same distribution. The *P*-values at least partially normalize for the sensitivity of the shape divergences to the number of neighbors. The results confirm the quality of the models for mitochondria and nucleoli and the higher quality of the Vox2Vox-RU Golgi model compared to the U-Net Golgi model.

Another question we addressed is whether there is a relationship between the shape and position of subcellular organelles. We fit a simple linear regression with organelles’ position as explanatory variables and the first principle component of their corresponding SPHARM descriptors as response. The resulting *R*^2^ scores were 0.0158, 0.0092, 0.08 and 0.0077 for nucleoli, Golgi apparatus, mitochondria and desmosome, respectively, suggesting a lack of correlation between organelle shape and position (at least for these organelles).

## 4 Discussion

In this article, we introduce new approaches for evaluating the quality of generative models of subcellular organelles. The first is based upon the expectation that individual organelles are largely spatially distinct from each other. To measure the extent to which generative models meet this expectation, we have introduced measures of exclusivity in which we compare the predictions for different proteins from the same transmitted light image and measure the overlap among different organelles at the same pixel position. High values of exclusivity were observed for a number of organelles. However, some organelles, such as microtubules, actin filaments and endoplasmic reticulum, showed fairly low values. Such low values can result from blurry predictions but can also reflect similarity between the appearance of different organelles in light microscope images (which results in the models predicting that both organelles appear at the same position). Such ambiguity is certainly to be expected, and the ability of some organelles to be predicted with high exclusivity is a remarkable feature of the approach developed by. Our criteria reflect which organelles are suited to that approach.

Given our finding that some organelle predictions overlapped significantly, we developed a retraining process that uses the predictions for other subcellular organelles to guide model learning for a particular organelle. This was done by adding a loss term that penalizes overlaps, and the results demonstrated that organelle exclusivity could be improved without major loss in reconstruction error. Future work may focus on exploring other choices of loss function to model the interdependencies among channels. We anticipate that our approach to object-wise representations of cell images can not only be used to evaluate different generative modeling approaches, but also to enable better modeling of phenotypic changes in subcellular organization resulting from perturbations or genetic differences.

Our second evaluation approach is based on the fact that many organelles exist as discrete objects. We therefore asked whether the shape and spatial distributions of the objects in synthetic images adequately reproduce those of the objects in real images. Using a proven approach for shape modeling, we demonstrated that generative models for at least some organelles do in fact produce objects similar to real images. To our knowledge, this is the first quantitative demonstration that this is the case.

We have also introduced an improved GAN-based pipeline for learning to predict realistic fluorescence microscope images for subcellular organelles from bright-field microscopy images. The synthetic images produced by our new method are better predictions by all of our criteria. Our proposed approach on object-wise metrics has demonstrated the practicality and efficiency on evaluating the morphology of subcellular structures. A possible direction for future work may be to bring the spirit of object-wise metrics into the construction of learning objectives or as part of the feedback in the learning process.

## Acknowledgements

We thank Gregory R. Johnson for helpful discussions.

## Funding

This work was supported in part by the U.S. National Institutes of Health [GM103712].

*Conflict of Interest*: none declared.

## Supplementary Material

btac688_Supplementary_Data
