# Supplementary material for: Improving and evaluating deep learning models of cellular organization
Source: Bioinformatics. 2022 Oct 20;38(23):5299–306. doi: 10.1093/bioinformatics/btac688 (PMC9710556; doi:10.1093/bioinformatics/btac688)
Supplement: btac688_Supplementary_Data [file btac688_supplementary_data.pdf]

Improving and evaluating deep learning models of cellular organization: Supplementary files

Huangqingbo Sun, Xuecong Fu, Serena Abraham, Shen Jin, and Robert F Murphy

October 11, 2022

1 Supplementary Tables and Figures

microtubule	$\mathcal{L}$ =GAN Loss + 25 × L2-Loss + 5 × Exclusivity Loss
actin filament	$\mathcal{L}$ =GAN Loss + 25 × L2-Loss + 5 × Exclusivity Loss
desmosome	$\mathcal{L}$ =GAN Loss + 10 × L1-Loss + 5 × Exclusivity Loss
DNA	$\mathcal{L}$ =GAN Loss + 10 × L1-Loss + 5 × Exclusivity Loss
nucleoli	$\mathcal{L}$ =GAN Loss + 10 × L1-Loss + 5 × Exclusivity Loss
nuclear envelope	$\mathcal{L}$ =GAN Loss + 25 × L1-Loss + 5 × Exclusivity Loss
cell membrane	$\mathcal{L}$ =GAN Loss + 25 × L1-Loss + 5 × Exclusivity Loss
actomyosin bundle	$\mathcal{L}$ =GAN Loss + 25 × L1-Loss + 5 × Exclusivity Loss
endoplasmic reticulum	$\mathcal{L}$ =GAN Loss + 10 × L2-Loss + 5 × Exclusivity Loss
Golgi apparatus	$\mathcal{L}$ =GAN Loss + 10 × L1-Loss + 5 × Exclusivity Loss
mitochondria	$\mathcal{L}$ =GAN Loss + 10 × L2-Loss + 5 × Exclusivity Loss
tight junction	$\mathcal{L}$ =GAN Loss + 10 × L2-Loss + 5 × Exclusivity Loss

Table S1: The loss functions used in retraining the Vox2Vox-RU model

	initial training			after retraining	
	U-Net	3D Pix2Pix	Vov2Vox-RU	U-Net	Vov2Vox-RU
microtubule	0.374	0.53	0.461	1.03	0.456
actin filament	0.439	0.596	0.606	0.763	0.662
desmosome	31.4	1.905	1.484	-	1.64
DNA	0.599	0.859	0.823	2.13	0.876
nucleoli	0.249	0.311	0.304	0.380	0.296
nuclear envelope	0.285	0.41	0.382	0.591	0.349
cell membrane	0.629	0.84	0.811	1.32	0.777
actomyosin bundle	0.767	1.027	0.942	0.855	1.37
endoplasmic reticulum	0.455	0.669	0.683	0.913	0.598
Golgi apparatus	0.882	1.476	1.40	0.948	1.76
mitochondria	0.513	0.722	0.620	0.550	0.697
tight junction	0.740	0.983	0.734	0.770	0.767
Overall (no desmosome)	0.5363	0.766	0.705	0.919	0.78
Overall	3.185	0.861	0.7691	3.535	0.856

Table S2: The MSE on the test set of the initial and retrained U-Net and Vox2Vox-RU models

Model	U-Net	U-Net retrained	Vox2Vox-RU	Vox2Vox-RU retrained	Ground-truth
	Mitochondria				
# objects	2538	2519	2659	2626	2591
Average Hausdorff distance	0.9985	1.0459	1.0152	1.087	1.0166
	Nucleoli				
# objects	357	349	416	380	386
Average Hausdorff distance	1.0439	1.0185	0.9951	1.0277	1.0136
	Golgi apparatus				
# objects	1156	1041	1508	1437	1211
Average Hausdorff distance	0.9122	0.9086	0.9484	0.9819	0.9522
	Desmosome				
# objects	-	-	629	867	751
Average Hausdorff distance	-	-	0.8670	0.8712	0.8658

Table S3: Number of subcellular objects found in the synthetic and real images. The average Hausdorff distance measures the quality of spherical harmonic transform modeling of the objects, lower is better.

Nucleoli				
Model	U-Net	U-Net retrained	Vox2Vox-RU	Vox2Vox-RU retraine
k=2				
Shape divergence	0.3624	0.3566	0.3235	0.3443
p-value	0.16	0.44	0.86	0.54
k=4				
Shape divergence	0.1558	0.1386	0.1293	0.1435
p-value	0.42	0.74	0.94	0.54
k=8				
Shape divergence	0.0716	0.06	0.0545	0.0547
p-value	0.28	0.8	0.94	0.96
k=16				
Shape divergence	0.03224	0.031	0.0227	0.0248
p-value	0.4	0.56	1	0.88
k=24				
Shape divergence	0.0232	0.0222	0.0154	0.0161
p-value	0.16	0.28	0.94	0.82

Golgi apparatus				
Model	U-Net	U-Net retrained	Vox2Vox-RU	Vox2Vox-RU retraine
k=2				
Shape divergence	0.3452	0.3398	0.3394	0.3398
p-value	0.58	0.74	0.74	0.74
k=4				
Shape divergence	0.1553	0.1705	0.1539	0.1509
p-value	0.36	<0.02	0.36	0.58
k=8				
Shape divergence	0.0713	0.0815	0.0684	0.0653
p-value	0.12	<0.02	0.38	0.7
k=16				
Shape divergence	0.0374	0.0425	0.0325	0.0321
p-value	0.06	<0.02	0.4	0.42
k=24				
Shape divergence	0.0265	0.0305	0.0229	0.0219
p-value	0.06	<0.02	0.24	0.36

Mitochondria				
Model	U-Net	U-Net retrained	Vox2Vox-RU	Vox2Vox-RU retraine
k=2				
Shape divergence	0.3435	0.3475	0.3430	0.3528
p-value	0.66	0.44	0.64	0.22
k=4				
Shape divergence	0.1521	0.1546	0.1519	0.1559
p-value	0.52	0.3	0.52	0.3
k=8				
Shape divergence	0.0679	0.0698	0.0664	0.0678
p-value	0.4	0.24	0.56	0.52
k=16				
Shape divergence	0.0319	0.0325	0.0320	0.0329
p-value	0.38	0.52	0.52	0.38
k=24				
Shape divergence	0.0207	0.0212	0.0213	0.0214
p-value	0.48	0.42	0.36	0.36

Desmosome		
Model	Vox2Vox-RU	Vox2Vox-RU retraine
k=2		
Shape divergence	0.3522	0.3411
p-value	0.46	0.68
k=4		
Shape divergence	0.1711	0.1625
p-value	0.1	0.2
k=8		
Shape divergence	0.0754	0.0748
p-value	0.16	0.14
k=16		
Shape divergence	0.0372	0.0353
p-value	0.1	0.18
k=24		
Shape divergence	0.0238	0.0226
p-value	0.12	0.28

Table S4: Divergence of objects in spherical harmonic descriptor shape space from real and synthetic images and their p-values.

Model	U-Net	U-Net retrained	Vox2Vox-RU	Vox2Vox-RU retrained
Mitochodria				
Spatial divergence	0.0004	0.0008	0.0025	0.0011
p-value	0.98	0.8	0.06	0.38
Nucleoli				
Spatial divergence	0.0007	0.0013	0.0005	0.0007
p-value	1	0.98	1	1
Golgi apparatus				
Spatial divergence	0.0073	0.0053	0.0014	0.0029
p-value	<0.02	0.04	0.98	0.4
Desmosome				
Spatial divergence	-	-	0.1893	0.3045
p-value	-	-	<0.02	<0.02

Table S5: KL divergence in subcellular spatial distributions between real and synthetic images.

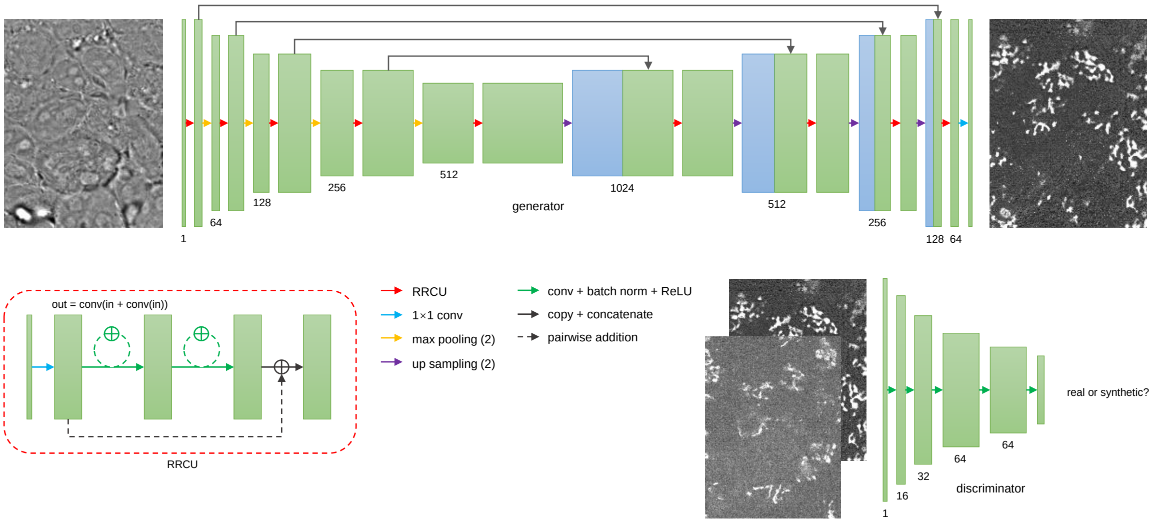

Figure S1: Neural network architecture of our proposed Vox2Vox-RU network.

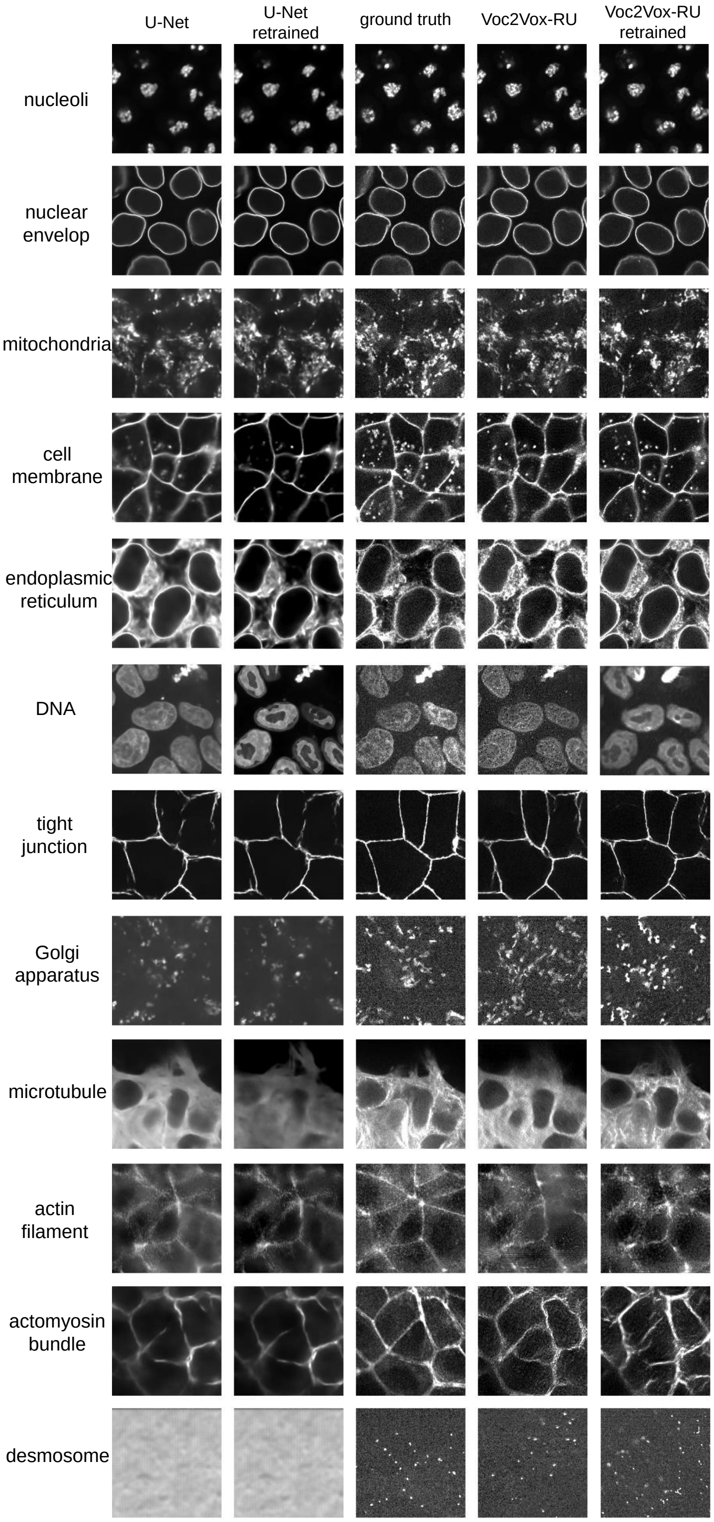

Figure S2: Example fluorescence tag predictions from U-Net (1st column), retrained U-Net (2nd column), real images (3rd column), and Vox2Vox-RU (4th column), and retrained Vox2Vox-RU (5th column). Note that desmosome is not retrained with U-Net, therefore the 1st column and 2nd column of the last row remain the same.

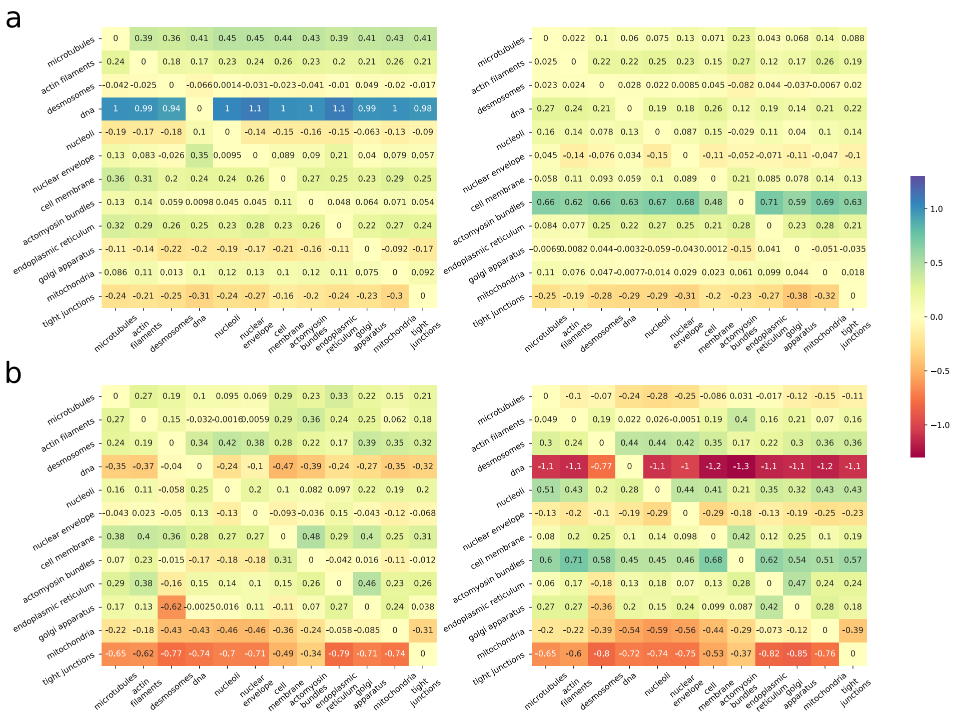

Figure S3: (a) The element-wise differences between the pairwise exclusivities of predictions from models before and after retraining of U-Net (left) and Vox2Vox-RU (right). (b) The element-wise differences between the pairwise exclusivities of predictions from Vox2Vox-RU and from U-Net models before (left) and after (right) retraining

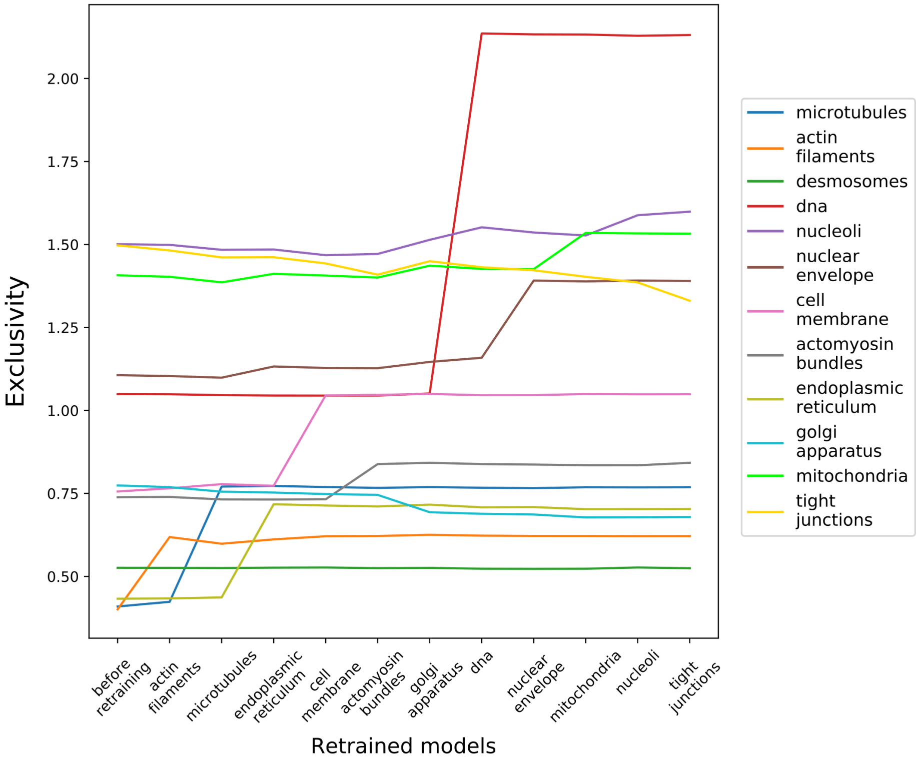

Figure S4: Individual organelle exclusivity changes during the retraining of U-Net models. The x-axis shows the organelle names in the retraining order and the lines with different colors show the individual organelle exclusivity changes.

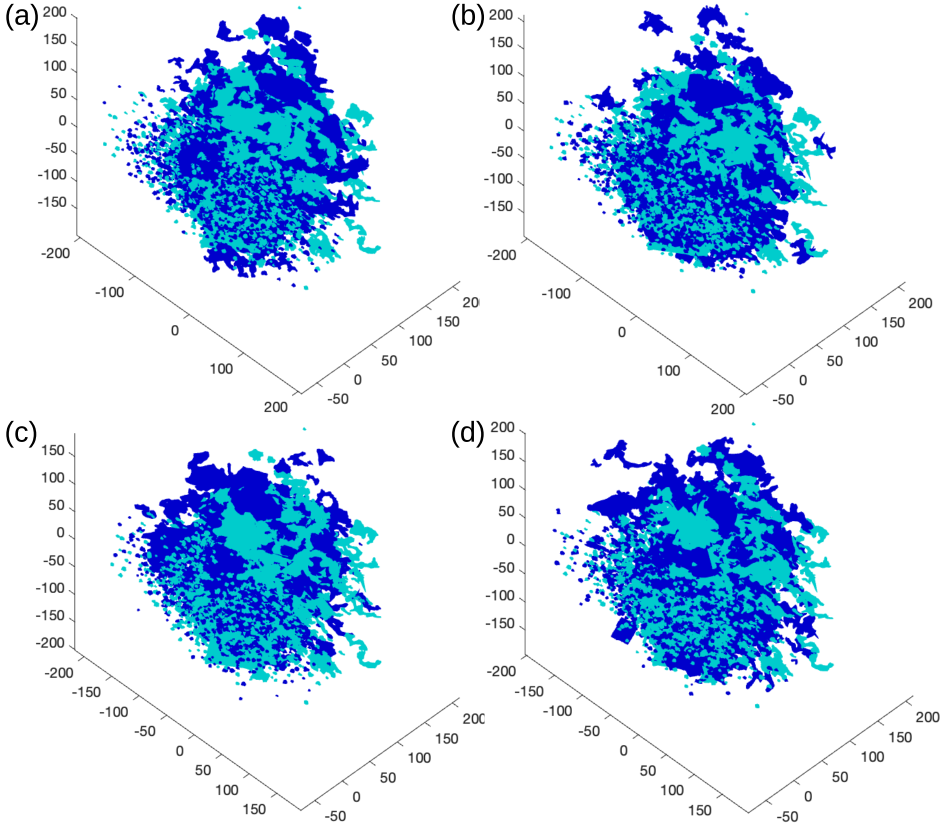

Figure S5: Visualization of mitochondria object shapes in the reduced spherical harmonic descriptor space. The light blue objects are from real images; the dark blue objects are from initial (a) and retrained (b) U-Net model without retraining and from initial (c) and retrained (d) Vox2Vox-RU model.

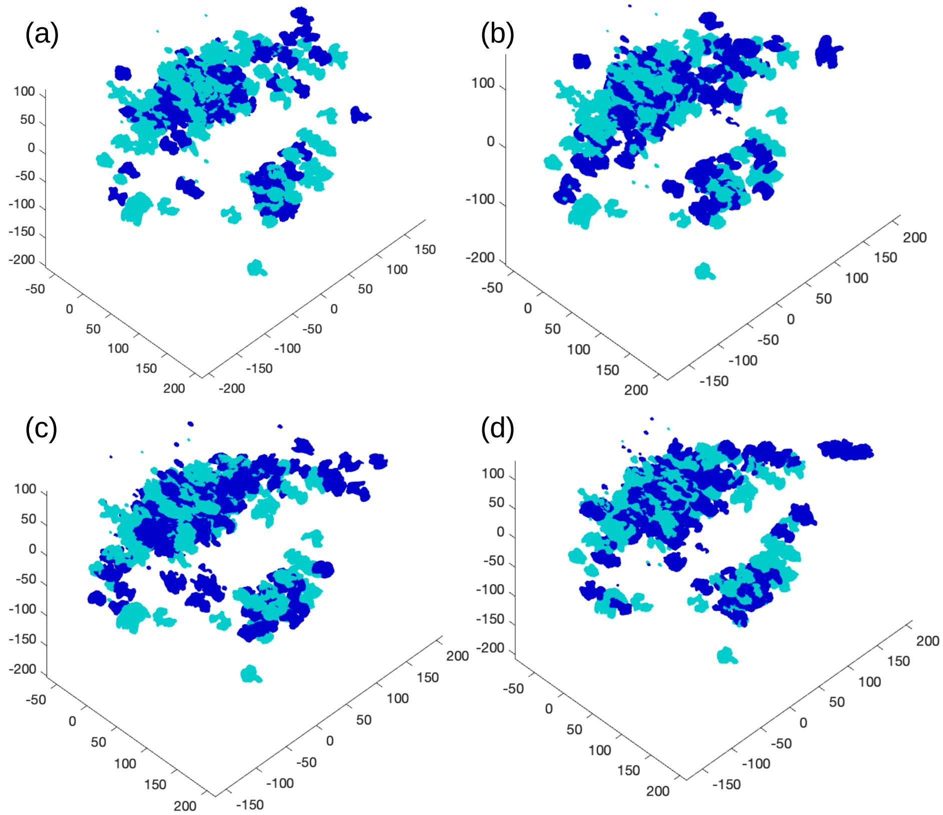

Figure S6: Visualization of nucleoli object shapes in the reduced spherical harmonic descriptor space. The light blue objects are from real images; the dark blue objects are from initial (a) and retrained (b) U-Net model without retraining and from initial (c) and retrained (d) Vox2Vox-RU model.

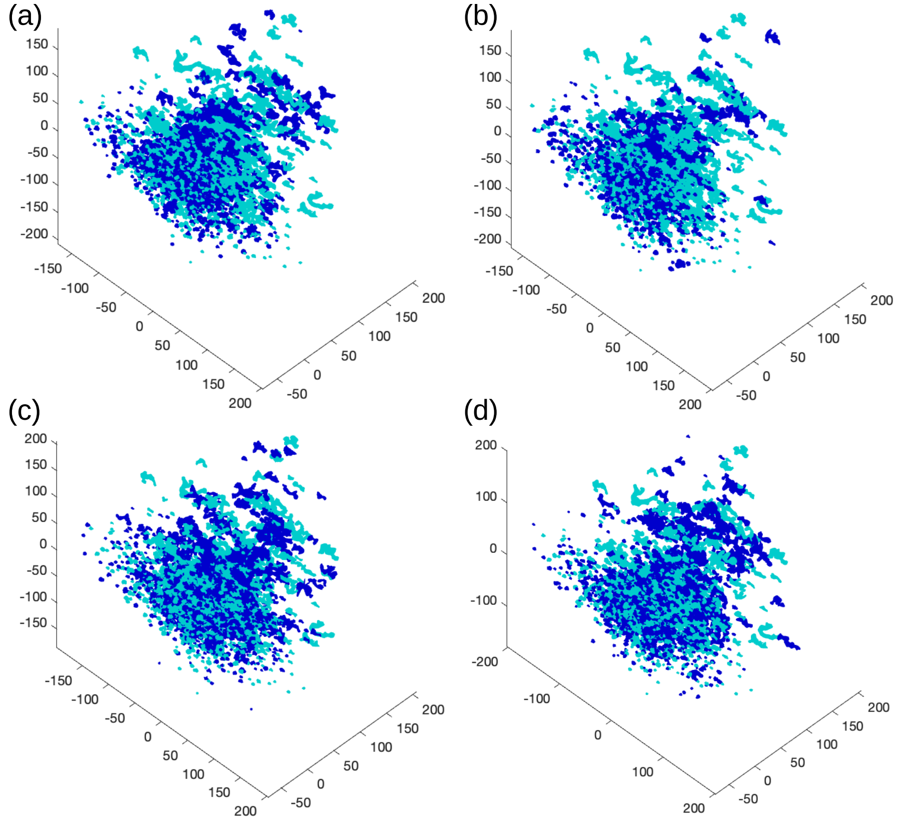

Figure S7: Visualization of Golgi apparatus object shapes in the reduced spherical harmonic descriptor space. The light blue objects are from real images; the dark blue objects are from initial (a) and retrained (b) U-Net model without retraining and from initial (c) and retrained (d) Vox2Vox-RU model.

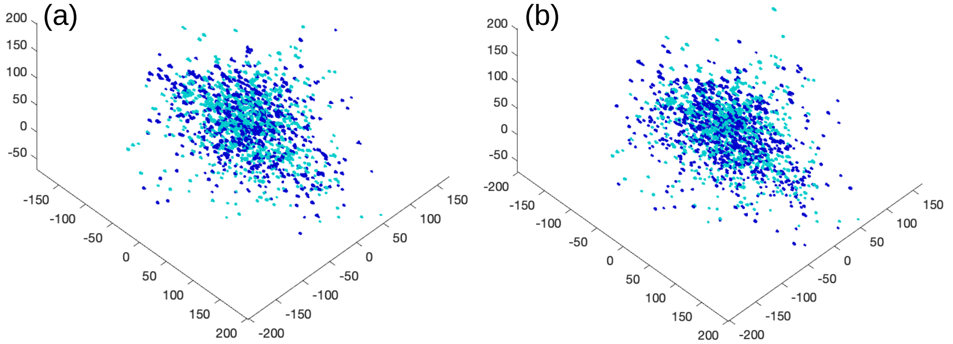

Figure S8: Visualization of desmosome object shapes in the reduced spherical harmonic descriptor space. The light blue objects are from real images; the dark blue objects are from initial (a) and retrained (b) Vox2Vox-RU model.Reduced spherical harmonic descriptor space of desmosome objects.

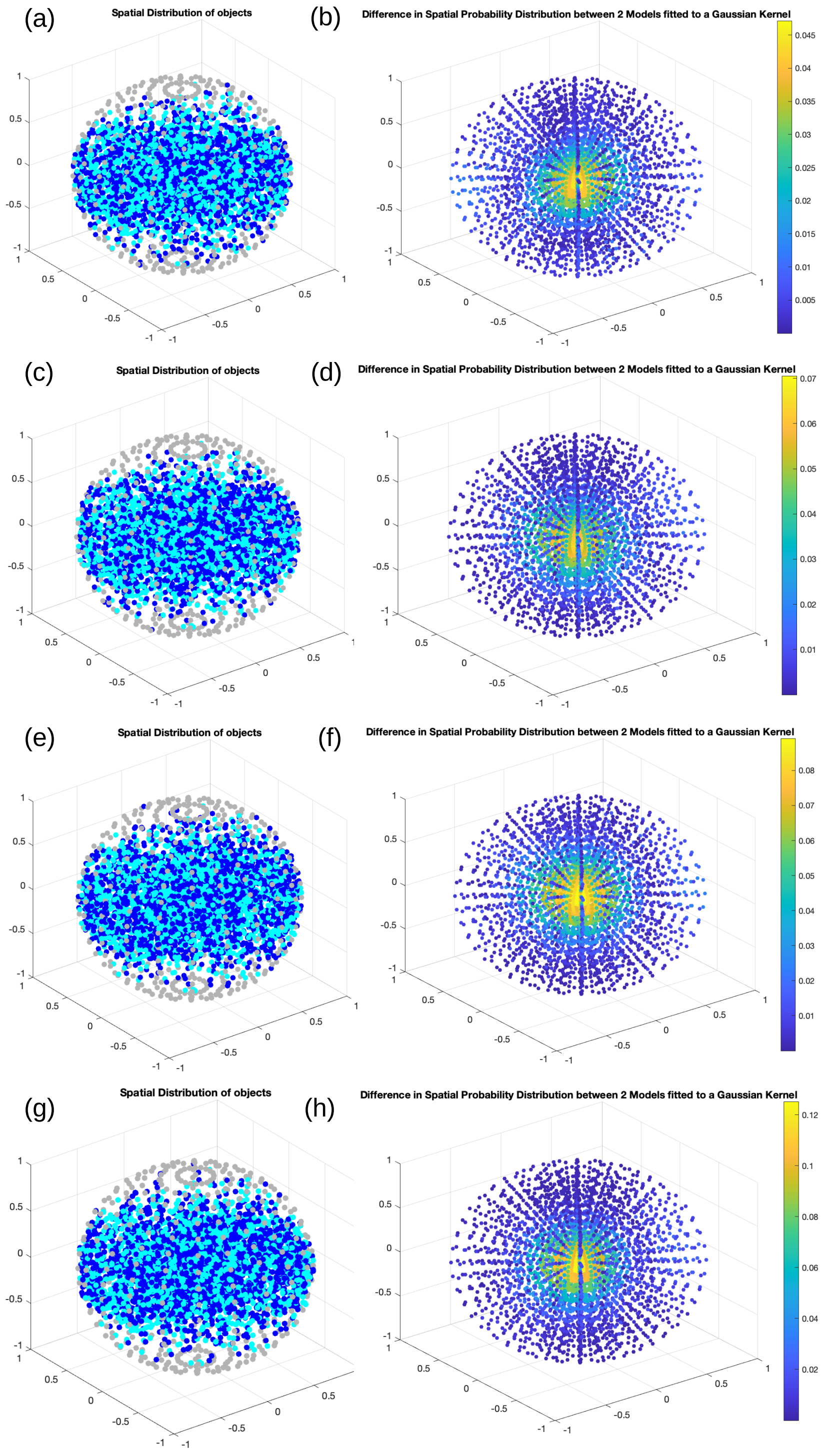

Figure S9: Spatial distribution analysis of mitochondria objects. Object positions are mapped onto a unit sphere whose center refers to the center of the cell and whose surface refers to the cell membrane. The light blue points are for objects from real images and the dark points refer to the objects from initial (a) and retrained (c) U-Net models and initial (e) and retrained (g) Vox2Vox-RU models. The absolute difference of the Gaussian smoothed spatial distributions between the objects from the real images and synthetic images are shown for the initial (b) and retrained (d) U-Net models and for the initial (f) and retrained (h) Vox2Vox-RU models.

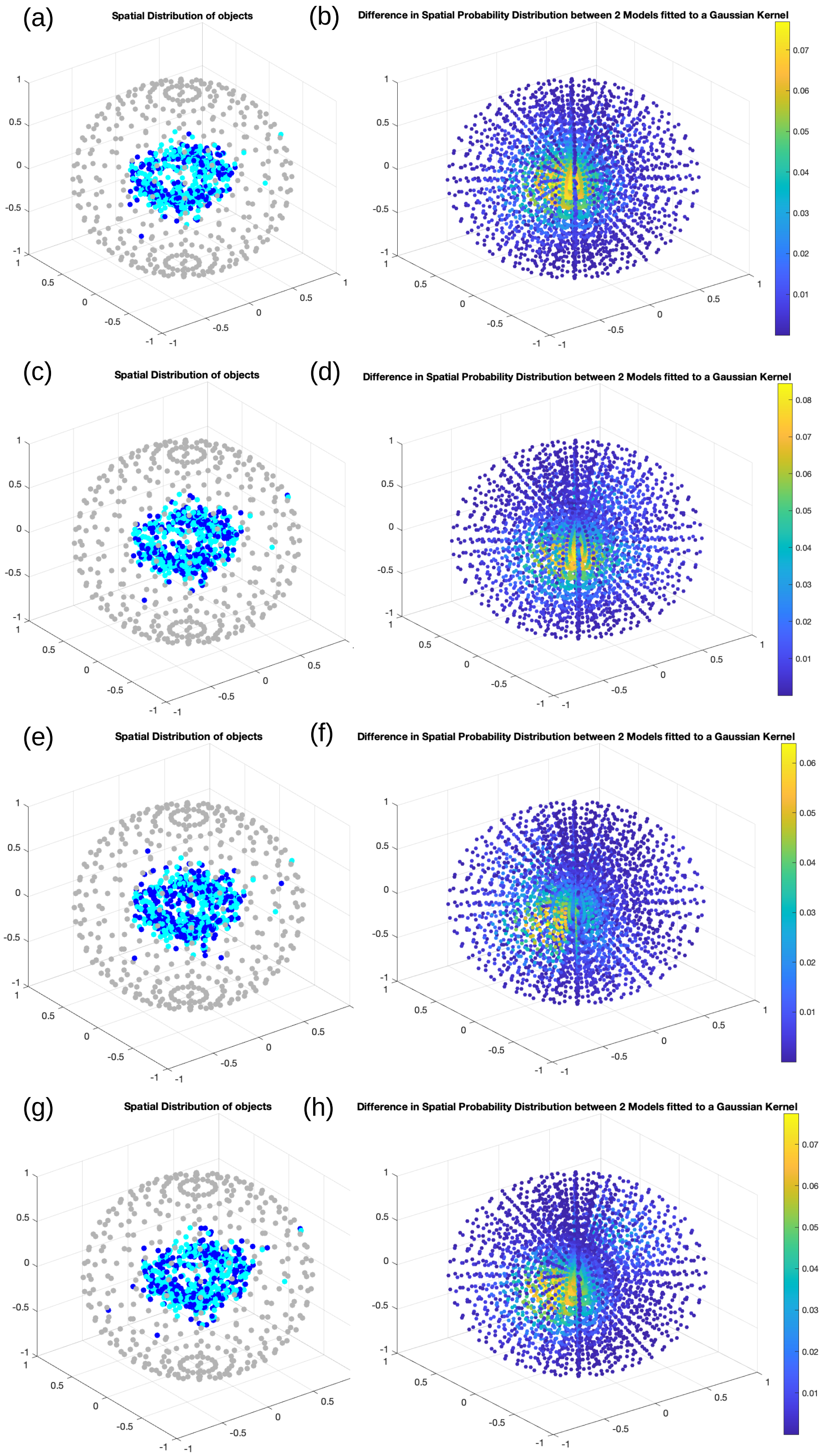

Figure S10: Spatial distribution analysis of nucleoli objects. Object positions are mapped onto a unit sphere whose center refers to the center of the cell and whose surface refers to the cell membrane. The light blue points are for objects from real images and the dark points refer to the objects from initial (a) and retrained (c) U-Net models and initial (e) and retrained (g) Vox2Vox-RU models. The absolute difference of the Gaussian smoothed spatial distributions between the objects from the real images and synthetic images are shown for the initial (b) and retrained (d) U-Net models and for the initial (f) and retrained (h) Vox2Vox-RU models.

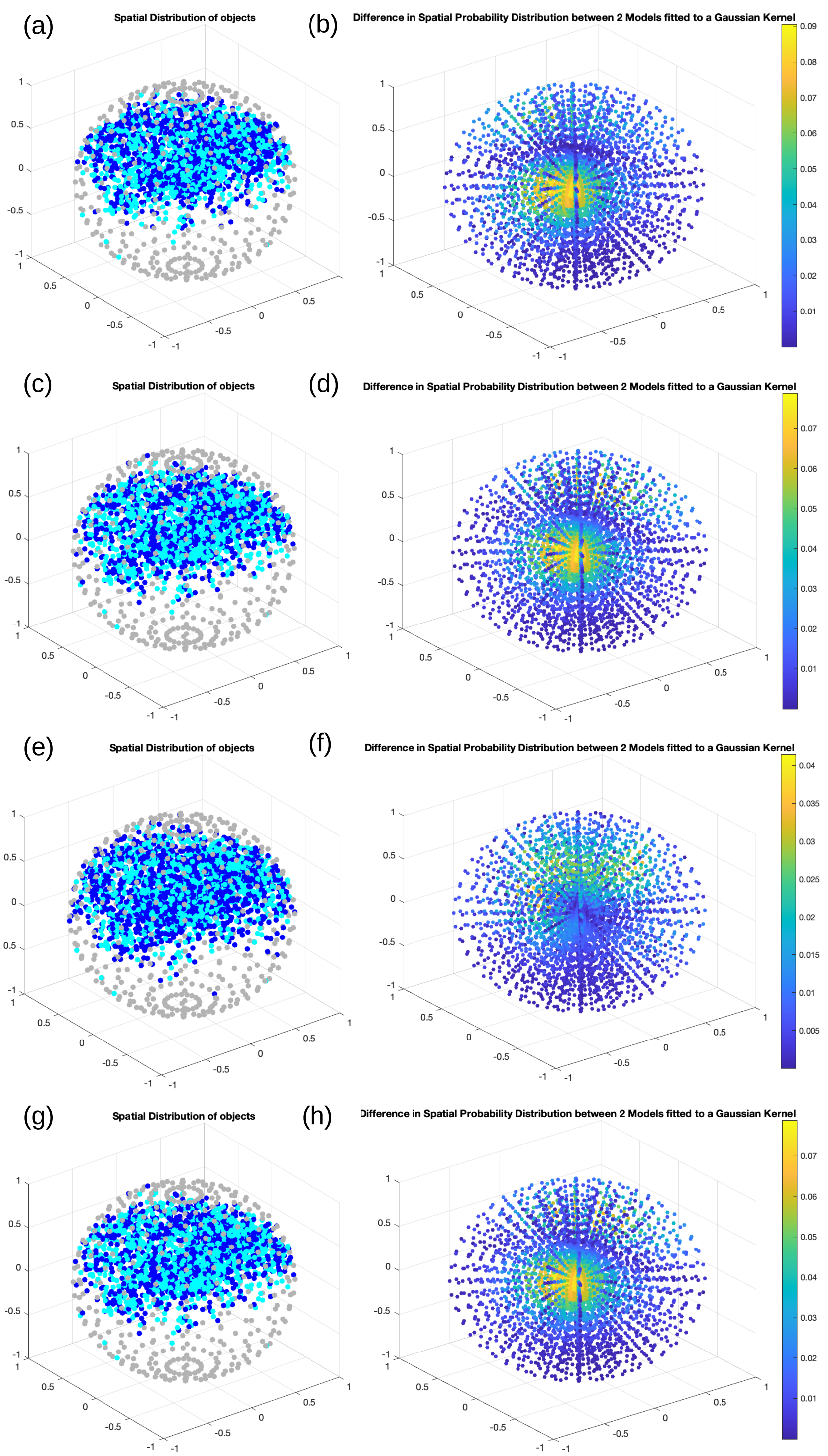

Figure S11: Spatial distribution analysis of Golgi apparatus objects. Object positions are mapped onto a unit sphere whose center refers to the center of the cell and whose surface refers to the cell membrane. The light blue points are for objects from real images and the dark points refer to the objects from initial (a) and retrained (c) U-Net models and initial (e) and retrained (g) Vox2Vox-RU models. The absolute difference of the Gaussian smoothed spatial distributions between the objects from the real images and synthetic images are shown for the initial (b) and retrained (d) U-Net models and for the initial (f) and retrained (h) Vox2Vox-RU models.

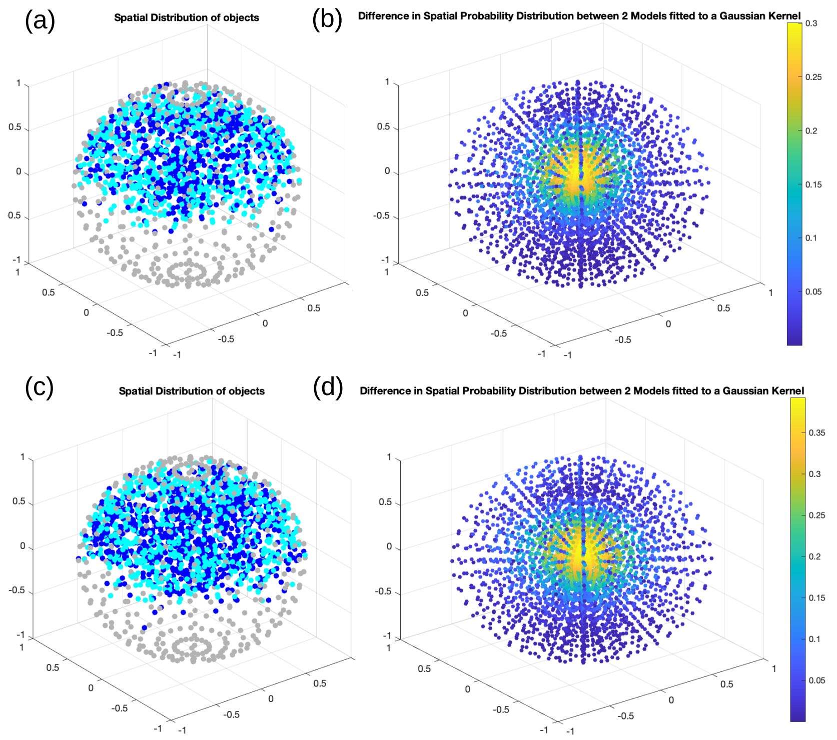

Figure S12: Spatial distribution analysis of desmosome objects. Object positions are mapped onto a unit sphere whose center refers to the center of the cell and whose surface refers to the cell membrane. The light blue points are for objects from real images and the dark points refer to the objects from initial (a) and retrained (c) Vox2Vox-RU models. The absolute difference of the Gaussian smoothed spatial distributions between the objects from the real images and synthetic images are shown for the initial (b) and retrained (d) Vox2Vox-RU models.
